# Increased extracellular matrix deposition during chondrogenic differentiation of dental pulp stem cells from individuals with neurofibromatosis type 1: an in vitro 2D and 3D study

**DOI:** 10.1186/s13023-018-0843-1

**Published:** 2018-06-25

**Authors:** Paula Nascimento Almeida, Deuilton do Nascimento Barboza, Eloá Borges Luna, Maria Clara de Macena Correia, Rhayra Braga Dias, Ana Caroline Siquara de Sousa, Maria Eugenia Leite Duarte, Maria Isabel Doria Rossi, Karin Soares Cunha

**Affiliations:** 1 Graduate Program in Pathology, School of Medicine, Universidade Federal Fluminense, Niterói, Rio de Janeiro Brazil; 2 Neurofibromatosis National Center (Centro Nacional de Neurofibromatose), Rio de Janeiro, Rio de Janeiro Brazil; 3 Oral and Maxillofacial Surgery, Antônio Pedro University Hospital, Universidade Federal Fluminense, Niterói, Rio de Janeiro Brazil; 4 Dentistry College, Universidade Federal Fluminense, Niterói, Rio de Janeiro Brazil; 5 National Institute of Traumatology and Orthopedics (Instituto Nacional de Traumatologia e Ortopedia), Rio de Janeiro, Rio de Janeiro Brazil; 6 Department of Pathology, School of Medicine, Universidade Federal Fluminense, Niterói, Rio de Janeiro Brazil; 7 Institute of Biomedical Sciences, and Clementino Fraga Filho University Hospital, Universidade Federal do Rio de Janeiro, Rio de Janeiro, Rio de Janeiro Brazil

**Keywords:** Neurofibromatosis 1, Chondrogenesis, Cell culture, Cell differentiation

## Abstract

**Background:**

Neurofibromatosis 1 (NF1) presents a wide range of clinical manifestations, including bone alterations. Studies that seek to understand cellular and molecular mechanisms underlying NF1 orthopedic problems are of great importance to better understand the pathogenesis and the development of new therapies. Dental pulp stem cells (DPSCs) are being used as an in vitro model for several diseases and appear as a suitable model for NF1. The aim of this study was to evaluate in vitro chondrogenic differentiation of DPSCs from individuals with NF1 using two-dimensional (2D) and three-dimensional (3D) cultures.

**Results:**

To fulfill the criteria of the International Society for Cellular Therapy, DPSCs were characterized by surface antigen expression and by their multipotentiality, being induced to differentiate towards adipogenic, osteogenic, and chondrogenic lineages in 2D cultures. Both DPSCs from individuals with NF1 (NF1 DPSCs) and control cultures were positive for CD90, CD105, CD146 and negative for CD13, CD14, CD45 and CD271, and successfully differentiated after the protocols. Chondrogenic differentiation was evaluated in 2D and in 3D (pellet) cultures, which were further evaluated by optical microscopy and transmission electron microscopy (TEM). 2D cultures showed greater extracellular matrix deposition in NF1 DPSCs comparing with controls during chondrogenic differentiation. In semithin sections, control pellets hadhomogenous-sized intra and extracelullar matrix vesicles, whereas NF1 cultures had matrix vesicles of different sizes. TEM analysis showed higher amount of collagen fibers in NF1 cultures compared with control cultures.

**Conclusion:**

NF1 DPSCs presented increased extracellular matrix deposition during chondrogenic differentiation, which could be related to skeletal changes in individuals with NF1.

**Electronic supplementary material:**

The online version of this article (10.1186/s13023-018-0843-1) contains supplementary material, which is available to authorized users.

## Background

Neurofibromatosis 1 (NF1; OMIM 162200) is an autosomal dominant syndrome caused by mutations in the *NF1* gene, located at chromosome 17q11.2, and affects 1:2000 individuals worldwide [1]. Neurofibromin, the *NF1* gene product, is a GTPase-activating protein (GAP) that interacts with Ras protein, which is involved in many signaling pathways that control cell proliferation, differentiation, and apoptosis. Neurofibromin converts Ras from an active (Ras-GTP) to an inactive form (Ras-GDP) [2]. Therefore, mutations in the *NF1* gene lead to an increased intracellular Ras-activity [3].

NF1 has a wide range of clinical manifestations, such as multiple neurofibromas, café-au-lait macules, Lisch nodules, and orthopedic problems (e.g. scoliosis, pseudoarthrosis of the tibia, dysplasia of long bones and sphenoid wing, and short stature) [4–6]. Skeletal manifestations in NF1 are mainly located in bones originated from endochondral ossification. Since chondroblasts express neurofibromin, [7] *NF1* mutations in chondroblasts may be important for the occurrence of orthopedic problems in NF1 individuals.

Animal models have been used to better understand the molecular mechanisms and pathogenesis of bone alterations in NF1, which is essential for the development of new therapies [8–10]. However, animal models have certain limitations to reproduce the skeletal manifestations of individuals with NF1 [11].

In vitro models are an alternative to study the clinical manifestations of NF1 and human stem cells cultures are already being used for this purpose [4, 12, 13]. Allouche et al. [12] used melanocytes derived from human embryonic stem cells as a model to study *café-au-lait* macules of NF1. Leskela et al. [4] used bone marrow mesenchymal stem cells obtained from NF1 children with orthopedic problems to investigate their osteogenic potential. There is only one previous study that explored the use of dental pulp stem cells (DPSCs) as an in vitro model to study NF1 [13]. In this study, Almeida et al. [13] proposed the use of DPSCs of deciduous teeth from individuals with NF1 to study the osteogenic differentiation alterations.

Dental stem cells are ectomesenchymal stem cells originated from the neural crest [14]. One advantage of using dental stem cells as in vitro models is their easy obtainment. There are five different sources of origin of dental stem cells: DPSCs [15]; stem cells from human exfoliated deciduous teeth (SHED) [16]; stem cells from periodontal ligament [17]; stem cells from dental follicle [18] and stem cells from apical papilla [19]. Considering the importance of studying chondroblasts to better understand the orthopedic problems that occur in NF1, the aim of this study was to evaluate the in vitro chondrogenic differentiation process of DPSCs from individuals with NF1 using both two-dimensional (2D) and three-dimensional (3D) cultures.

## Methods

### Subject selection

This study was conducted at Antônio Pedro University Hospital of Universidade Federal Fluminense, Niterói, Brazil, between 2013 and 2016, and was approved by the institution’s Ethics Committee (No.519.858/2014). All participants signed a consent form. DPSCs were obtained from human third molars, which were extracted following clinical recommendation. Two primary cell cultures were obtained from individuals with NF1 (NF1 group; NF37 and NF87) and three from individuals without NF1 (control group; CT10, CT11 and CT12). Participants were both men and women with ages ranging from 19 to 38 years.

### Isolation and cell culture

Immediately after extraction, each tooth was washed with sterile saline solution. Using a cylindrical Zekrya drill (Microdont, SP, BR), a groove was made at the amelocemental junction to facilitate the cleavage of the tooth with an orthodontic wire cutter. The pulp tissue was removed and transported to the culture laboratory in a conical tube containing 5 mL of DMEM-F12 medium (Dulbecco’s Modified Eagle Medium Nutrient Mixture F-12; Invitrogen, CA, USA), supplemented with 15% (*v*/v) fetal bovine serum (FBS) (HyClone™, Utah, USA), 100 U/mL penicillin and 100 μg/mL streptomycin (Invitrogen).

Pulp cell cultures were established by explant culture technique. The pulp tissue was placed in a 35 × 10 mm cell culture dish (Corning, NY, USA) containing 2 mL of DPSCs culture medium composed of DMEM-F12 medium supplemented with 12% (*v*/v) FBS, 100 U/mL penicillin and 100 μg/mL streptomycin, 2 mM L-glutamine (Invitrogen) and 0,01 mM non-essential amino acids (Invitrogen). Medium was changed every two days. At each passage, cells were washed three times with phosphate-buffered saline (PBS) and released from culture surface using trypsin-EDTA 0,25% (Gibco, NY, USA) for 5 min at 37 °C. DMEM-F12 medium supplemented with 12% (v/v) FBS was added to the cell suspension, and the cell number was determined using Countess II FL Automated Cell Counter (ThermoFisher Scientific, MA, USA). The cell suspension was then centrifuged and the pellet suspended in DPSCs culture medium. Cells were plated at a density of 1 × 10^4^ cells/cm^2^.

### Dental pulp stem cells characterization

In 2006, the International Society for Cellular Therapy proposed the minimum criteria to define mesenchymal stromal cells (MSCs), as follows: plastic adherence, specific surface antigen expression, and multipotent differentiation potential, including adipogenesis, osteogenesis and chondrogenesis [20]. These criteria were fulfilled in this study as follows.

### Surface antigens expression

At passage 3–4, 3 × 10^5^ cells were washed with PBS containing 0,1% sodium azide and 3% FBS and then incubated for 30 min in ice and in the dark with the following mouse anti-human monoclonal antibodies directly conjugated with fluorochromes: CD13/FITC (22A5, Caltag Medsystems, UK), CD14/PE (M5E2, BD Biosciences, CA, USA), CD34/FITC (581, BD Biosciences), CD45/ALexa405 (HI30, Caltag Medsystems), CD90/PECy5 (5E10, BioLegend, CA, USA), CD105/APC (45A5A3, BioLegend), CD146/PE (P1H12, BD Biosciences), CD271/APC (C401457, BD Biosciences). Unstained cells were used as controls. Data was acquired using FACSCanto II (BD Bioscienses) flow cytometer and at least 20.000 events were analyzed with FACSDiva (BD Biosciences) or FlowJo 7.6.5 (FlowJo.com; Tree Star, OR, USA) software.

### Adipogenic differentiation

For adipogenic differentiation, a triplicate of 5 × 10^3^ cells (passage 3) were plated in a 6-well culture dish and cultivated in DPSCs culture medium for two days. Medium was replaced by DMEM-High Glucose medium (Invitrogen) supplemented with 10% (*v*/v) FBS, 100 U/mL penicillin, 100 μg/mL streptomycin, 0,5 mM isobutyl methyl xanthine (Sigma-Aldrich, MO, USA), 10^− 6^ M dexamethasone, 10 μM insulin (Biobras, MG, BR) and 200 μM indomethacin (Sigma-Aldrich). Culture was kept for 21 days and medium was changed every three days.

To demonstrate the potential of adipogenic differentiation, cultures were fixed with 4% formaldehyde for 30 min and stained with Oil Red O. Plates were washed twice with distilled water, and 1 mL of propylene glycol PA was added for two minutes. After removing propylene glycol, plates were incubated with Oil Red O for 20 min at room temperature. Oil Red O was removed and 1 mL propylene glycol 85% was added for a minute. Plates were washed twice in distilled water and kept open until dry.

### Osteogenic differentiation

To induce osteogenic differentiation, a triplicate of 5 × 10^3^ cells (passage 3) were plated in a 6-well culture dish and cultivated in DPSCs culture medium for two days. Medium was then replaced by DMEM-High Glucose medium supplemented with 10% (*v*/v) FBS, 100 U/mL penicillin, 100 μg/mL streptomycin, 50 μM ascorbic acid 2-phosphate (Sigma-Aldrich, MO, USA), 10^− 8^ M dexamethasone (Sigma-Aldrich, MO, USA) and 10 mM β-glycerophosphate (Sigma-Aldrich, MO, USA). Culture was kept for 21 days and medium was changed every three days.

To demonstrate the potential of osteogenic differentiation, cultures were fixed with 4% formaldehyde for 30 min and stained with von Kossa method. Plates were washed twice with distilled water and incubated with 2% AgNO3 solution for one hour at room temperature in the dark. Dye excess was removed, and plates were rinsed with distilled water and exposed to ultraviolet light for 40 min.

### Chondrogenic differentiation

The induction of chondrogenic differentiation was performed following two protocols: 2D cell culture and 3D cell culture (pellet). For the 2D cell culture, 10^4^ cells (at passage 3; in quadruplicate) were plated with10 μL DMEM-High glucose medium in the center of a 24-well culture dish (Corning, NY, USA) and left in incubator for two hours at 37 °C. Following, 500 μL of DMEM-High glucose medium supplemented with 10% (*v*/v) FBS, 100 U/mL penicillin and 100 μg/mL streptomycin was added and the plate was incubated at 37 °C for 24 h. This medium was then supplemented with 6.25 μg/mL insulin and transferrin (Invitrogen, NY, USA), 10^− 8^ M dexamethasone,50 μM ascorbic acid 2-phosphate (added to medium only after the sixth day) and 10 μg/mL transforming growth factor beta 1 (TGFβ-1; Sigma-Aldrich, MO, USA). Culture was kept for 21 days and medium was changed every three days.

To demonstrate the potential of chondrogenic differentiation, cultures were fixed with 4% formaldehyde for 30 min, washed twice with distilled water and incubated with Alcian blue (pH 2.5) for 30 min at room temperature. Dye excess was removed and plates were rinsed with distilled water and kept open until dry.

For the 3Dcell culture, 5 × 10^5^ cells (at passage 3; in duplicate) were centrifuged in 15 mL polypropylene conical tubes at 1500 rpm (201,24 g) for five minutes. The resulting pellets were maintained in DMEM-High glucose medium supplemented with 10% (*v*/v) FBS and 100 U/mL penicillin and 100 μg/mL streptomycin. After 24 h, 6.25 μg/mL insulin and transferrin, 10^− 8^ M dexamethasone, 50 μM ascorbic acid 2-phosphate and 10 μg/mL TGFβ-1 were added to the culture medium. Cultures were kept for 21 days and medium was changed every three days. At the end of chondrogenic differentiation, all pellets were washed with PBS and fixed either with paraformaldehyde 4% (v/v) overnight for light microscopic analysis or with 2.5% glutaraldehyde solution with 0.1 M sodium cacodylate buffer for transmission electron microscopy (TEM).

### Quantitative analysis of chondrogenic differentiation in bidimensional cultures

After chondrogenic differentiation, one picture (20× magnification) was obtained per quadruplicate at the center of the culture dish. Images were analyzed using ImageJ software v.1.49 (NIH, USA), and the percentage of stained area in each picture was obtained.

### Pellet analysis after chondrogenic differentiation

For the analysis of chondrogenic differentiation in 3D pellet culture, paraffin sections with 3 μm thickness were stained with hematoxylin and eosin (HE) to evaluate pellet morphology, as well as with Alcian blue (pH 2.5) to evaluate glycosaminoglycans (GAGs) deposition. Expression of Ki-67 was evaluated immunohistochemically using mouse monoclonal antibody anti-Ki-67 (1:80; M7240; Dako Corporation, CA, USA). Glass slides were scanned with Aperio System (Leica Biosystems, CA, USA) at 40× and the results were descriptive.

For TEM, pellets were fixed with 1% osmium tetroxide for 1 h, dehydrated in ascending concentration of acetone and embedded in epoxy resin. Semithin sections with 0.5 μm were stained with 1% toluidine blue and scanned with Aperio System at 40× for descriptive analysis. Ultrathin sections (70 nm) were collected on copper grids and counterstained with 2% uranyl acetate and lead citrate. Images were obtained using JEM-1011 (JEOL, MA, USA). In the ultrastructure analysis, production of extracellular matrix was evaluated through the presence of collagen fibers and intra and extracellular vesicles.

### Statistical analysis

Statistical analysis was performed using Statistical Package for Social Sciences software (SPSS v. 20.0; Chicago, IL, USA) at 5% significance level. Proliferation and senescence assays were analyzed using repeated measure ANOVA with Bonferroni Correction, for comparison within groups. Student’s *t* test for independent groups was used for analyzing PDT and clonogenic assay results. For chondrogenic bidimensional differentiation analysis, Man-Whitney test was used.

## Results

### Dental pulp stem cells from NF1 individuals are phenotypically similar to control cells and retained adipogenic, osteogenic and chondrogenic differentiation potential

**Fig. 1:**
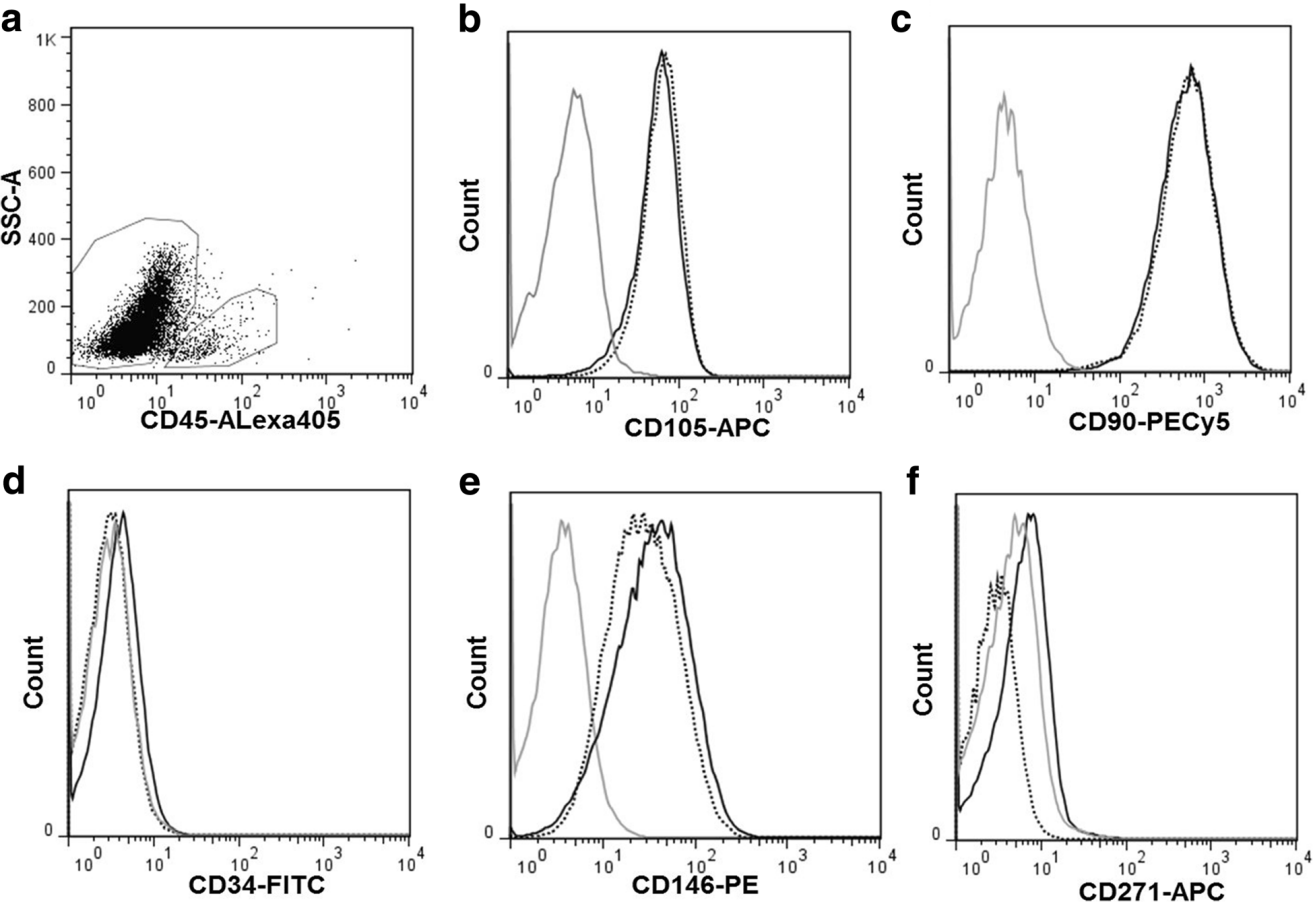
Flow cytometry of DPSCs from NF1 and control cultures at passage 3 in culture. **a**: Dot plot showing CD45/SSC complexity and fluorescence. The regions of CD45+ and CD45- cells are indicated. **b**-**f**: Histograms showing the expression of CD105, CD90, CD34, CD146 and CD271, respectively, in CD45- cells. Gray line: negative control; Black line: NF1 cultures and dotted line: control cultures

Both control and NF1 DPSCs at passage 3 and 4 showed a uniform expression of CD90, CD105 and CD146. All DPSCs cultures were negative for CD271 as well as for hematopoietic markers CD14 and CD34.The percentage of CD45+ cells was less than 5%, ranging from 1.7 to 4.0% (Fig. 1).

**Fig. 2:**
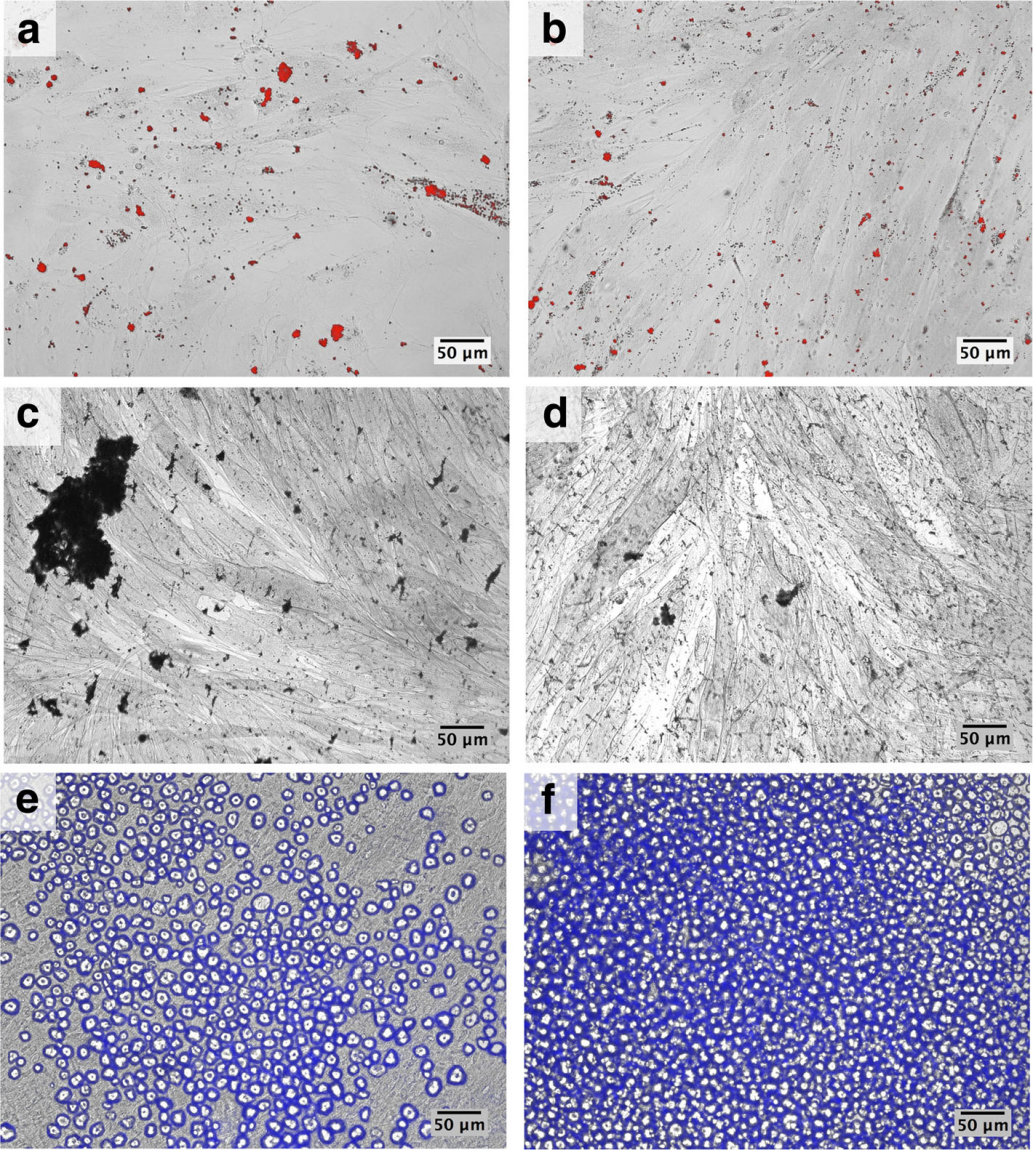
DPSCs were induced to differentiate towards adipogenic, osteogenic, and chondrogenic lineages in a 2D culture. Phase-contrast microscopy. **a**-**b**: Oil Red O staining shows lipids deposits in red after 21 days of adipogenic differentiation. **c**-**d**: von Kossa staining identifies calcium deposits at the end of osteogenic protocol. **e**-**f**: Alcian blue staining shows the presence of GAGs. A, C, E: CT12. B, D, F: NF87

The multipotency of DPSCs was demonstrated by their capacity to differentiate towards adipogenic, osteogenic and chondrogenic lineages. In all control and NF1 cell cultures, intracellular lipid droplets were observed after adipogenic differentiation (Fig. 2, a-b). In all cell cultures, after 21 days of osteogenic differentiation, the presence of calcium deposits was identified by von Kossa staining, although the amount of calcium deposition in NF1 cultures was reduced compared to control cultures (Fig. 2, c-d). At the end of chondrogenic differentiation, GAGs, a component of extracellular matrix, was identified by Alcian blue (Fig. 2, e-f).

### Extracellular matrix synthesis is increased during chondrogenic differentiation

**Fig. 3:**
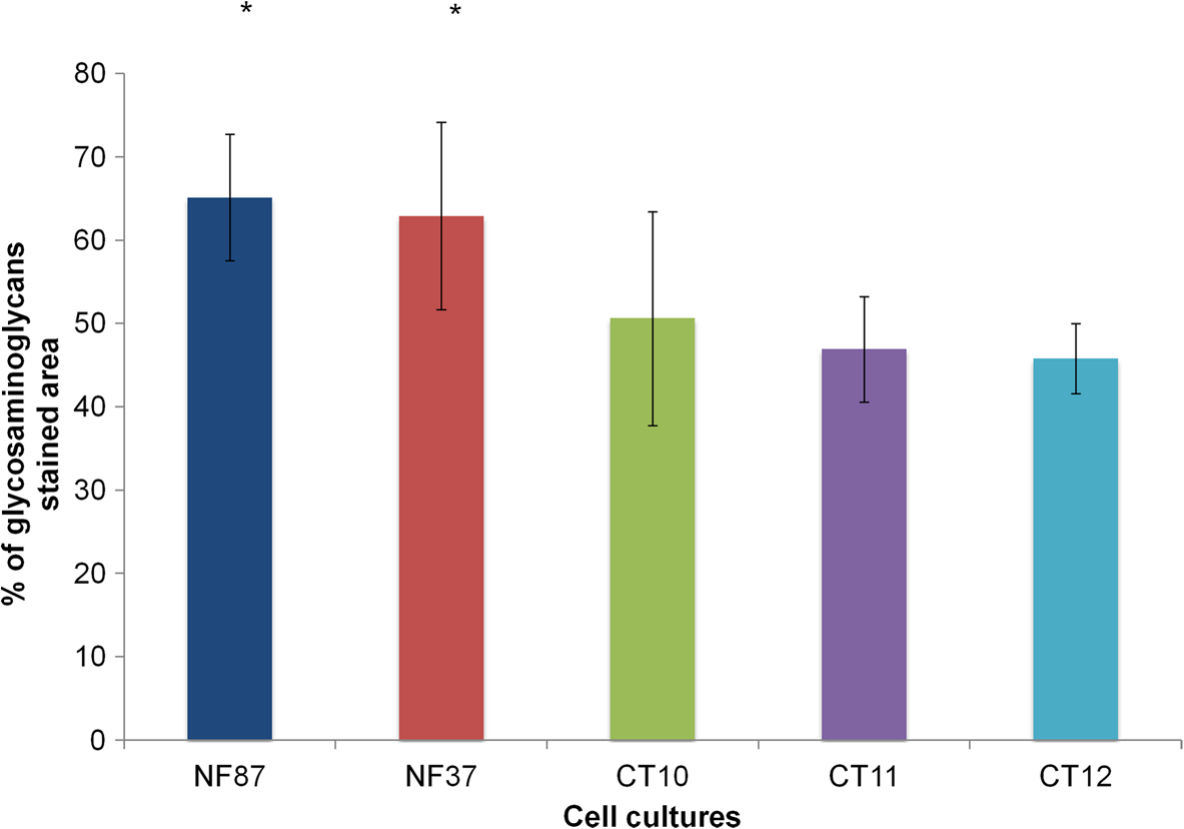
Percentage of GAGs stained area after chondrogenic differentiation in 2D cultures from NF1 and control cultures

**Fig. 4:**
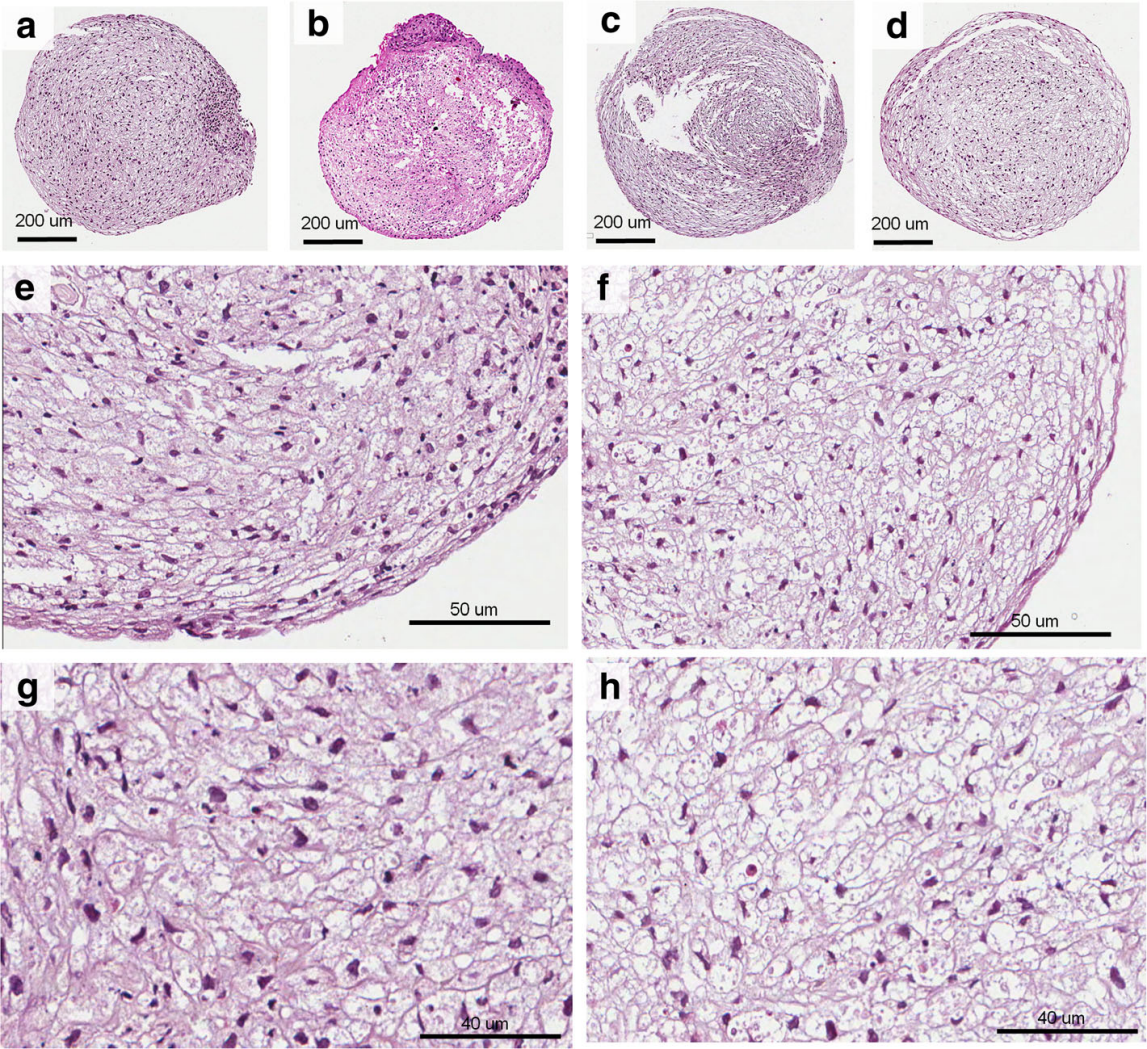
Control and NF1 pellets stained with hematoxylin and eosin after chondrogenic differentiation. **e**-**f**: Pellets with fusiform cells at the periphery. g-h: Pellets’ central area consisting of large, polygonal cells, with clear cytoplasm, reminding the morphology of chondroblasts. **a**, **e**, **g**: CT10. **b**: CT11. **c**: NF37. **d**, **f**, **h**: NF87

**Fig. 5:**
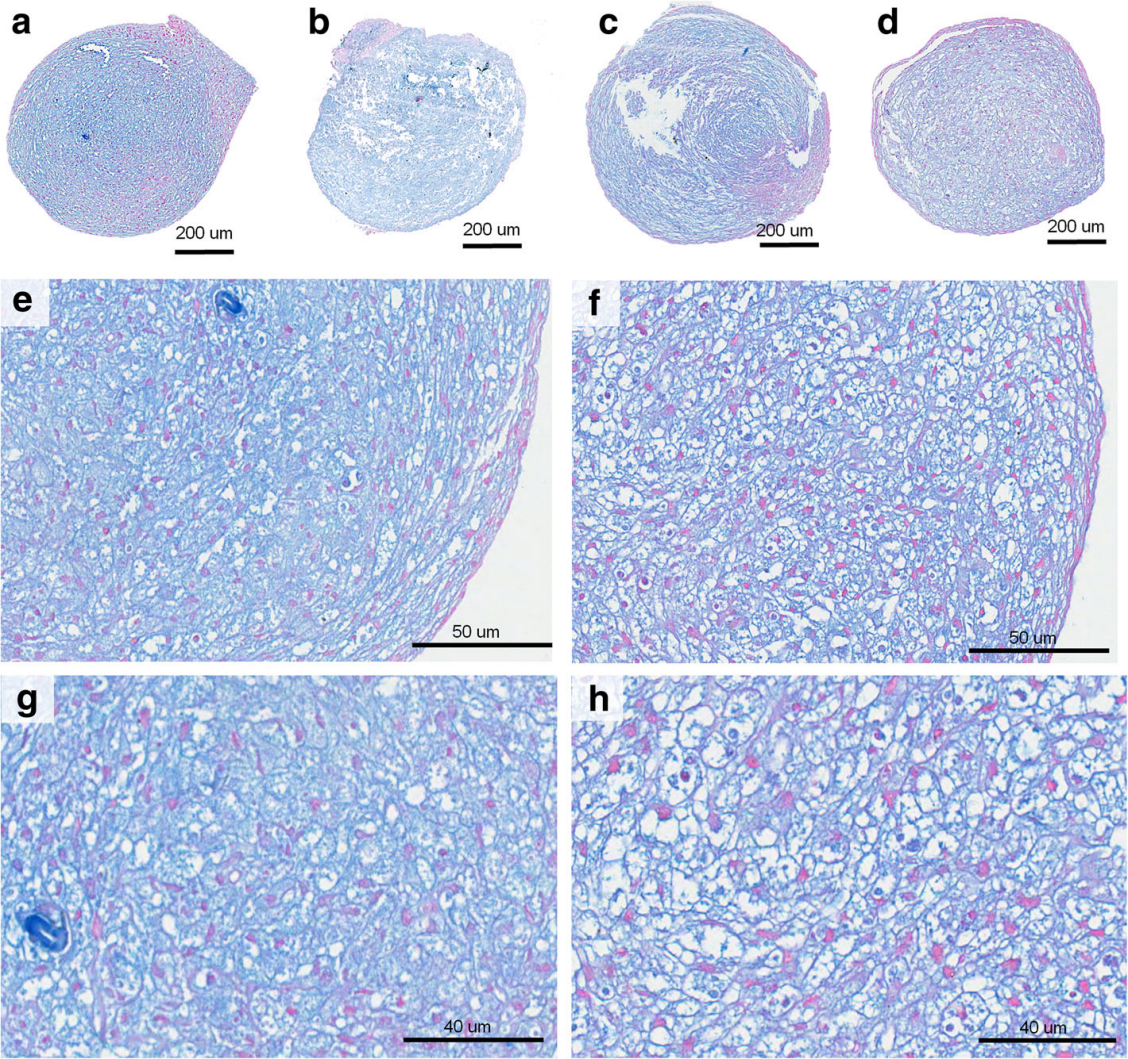
Control and NF1 pellets stained with Alcian blue after chondrogenic differentiation. GAGs are stained in blue. **e**, **j**: Cartilage-like fragment is seen. **a**, **e**, **g**: CT10. **b**: CT11. **c**: NF37. **d**, **f**, **h**: NF87

After chondrogenic differentiation in 2D cell cultures, the percentage of GAGs stained area was higher in the NF1 group compared with the control group (*p* = 0.004, Mann-Whitney test; Fig. 3). The quantification of the stained area is shown in Additional file 1. After 3D chondrogenic differentiation, pellets were macroscopically similar in all cultures, showing a reduction in size during the second week of differentiation and maintaining the size stable until the end of the protocol. In HE stained sections, one or two layers of fusiform cells were seen at the periphery of all pellets (Fig. 4, a-f). The other cells in the pellet were large, polygonal, with either granular or vesicular clear cytoplasm, and exhibited hyperchromatic nuclei, with a rounded, fusiform or irregular shape (Fig. 4, g-h). CT10, CT11 and NF37 pellets presented a polar area with a group of smaller cells with eosinophilic cytoplasm. Alcian blue staining confirmed the production of GAGs, indicating the success of chondrogenic differentiation (Fig. 5, a-h). None of the pellets showed Ki-67 expression, indicating that, at the 21st day of chondrogenic differentiation, there was no cell proliferation (see Additional file 2).

**Fig. 6:**
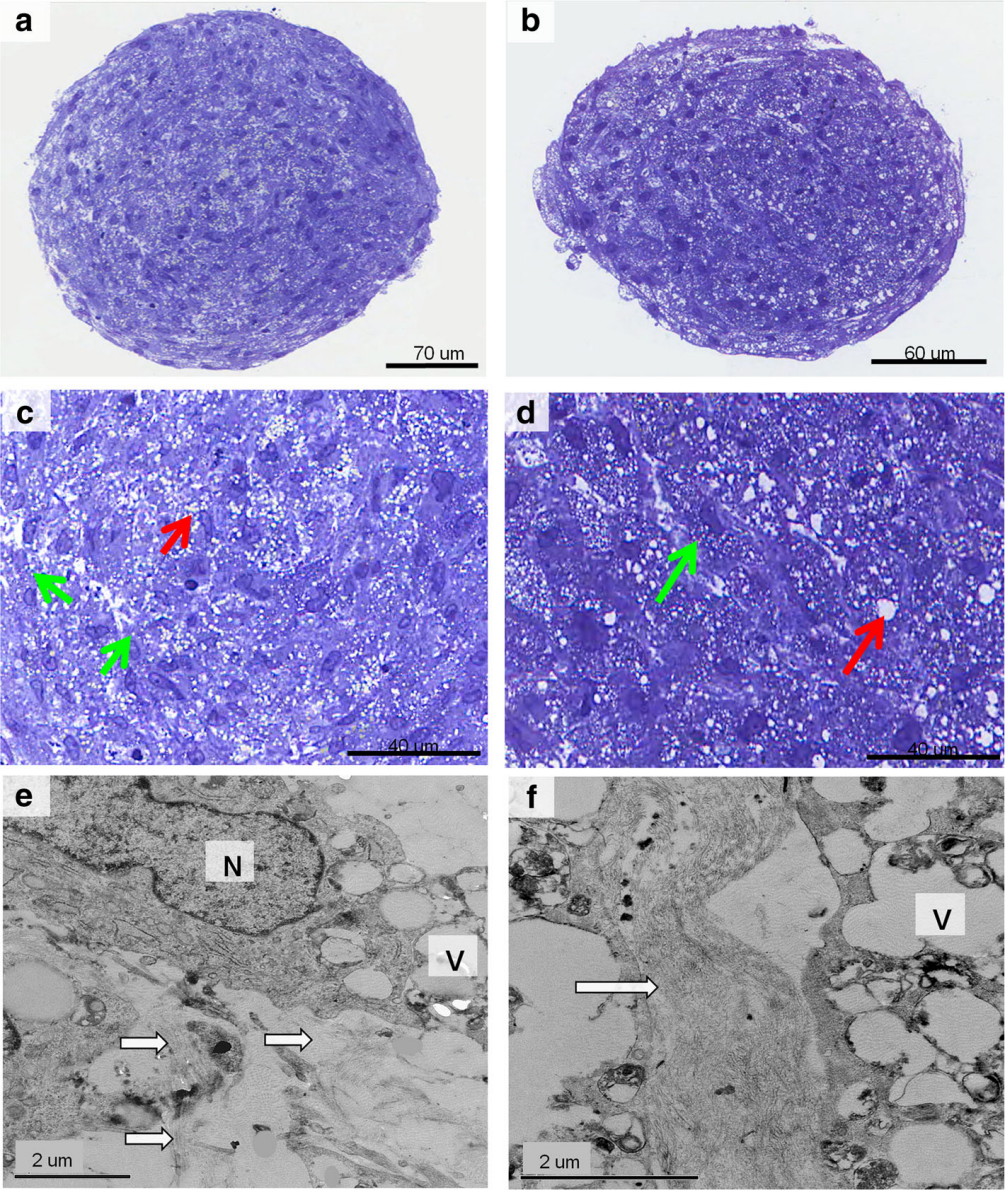
Ultrastructural analysis of control (**a**, **c**, **e**) and NF1 pellets (**b**, **d**, **f**) after chondrogenic differentiation. **a**-**d**: Semithin sections stained with toluidine blue. **c**-**d**: Intra and extracellular vesicles (green and red arrows, respectively). **c**: Control culture, vesicles are small and of uniform size. **d**: NF1 culture, vesicles present heterogeneity of sizes. **e**-**f**: V: matrix vesicles; N: nucleus; white arrows: collagen fibers

In control cultures, analysis of semithin sections showed small vesicles of homogeneous size distributed intra and extracellularly throughout the pellets. In NF1 cultures, there was heterogeneity in the size of vesicles, ranging from small to large sizes, located both intracellularly and extracellularly (Fig. 6, a-d). At the ultrastructural level, cells presented a chondrogenic phenotype, with rounded nuclei, predominantly euchromatic, and there was accumulation of matrix vesicles in the cytoplasm. In the extracellular space, collagen fibers were present in greater amount in NF1 cultures compared with control cultures (Fig. 6, e-f). In addition, NF1 cultures had larger and irregular extracellular matrix vesicles. A large amount of granular endoplasmic reticulum was present in both NF1 and control cultures, indicating metabolic activity.

## Discussion

To the best of our knowledge, this is the first study to characterize DPSCs from individuals with NF1 and evaluate their chondrogenic differentiation potential both in 2D and 3D cultures. A previous study proposed the use of SHED as an in vitro study model for NF1, but only osteogenic differentiation was performed [13]. To consider stem cells from dental origin as a potential in vitro model for studying NF1, a better characterization of those cells is mandatory. Therefore, we evaluated the expression of surface antigens and the multipotency as proposed by the International Society for Cellular Therapy for MSCs classification [20]. Since there are no specific surface markers for DPSCs, MSCs markers are typically used to characterize DPSCs [21–23]. In the present study, both control and NF1 cell cultures expressed CD90, CD105, and CD146. All cell cultures were negative (less than 5% of expression) for CD14, CD34, CD45, and CD271 markers. These results are similar to the ones reported in the literature from DPSCs from healthy individuals [23–25]. We showed that all control and NF1 cell cultures had the ability to undergo adipogenic, osteogenic and chondrogenic differentiation, which confirms their multipotency. Interestingly, the amount of calcium deposition in NF1 cultures after osteogenic differentiation was significantly lower compared to the control cultures, and this is similar to results reported by the previous study that used SHED (see Additional file 3) [13]. Regarding lipid deposition, no statistical difference between NF1 and control cultures was found after adipogenic differentiation (see Additional file 3).

Regarding chondrogenic differentiation, NF1 cell cultures had a higher percentage of stained GAGs in comparison with control cell cultures, indicating a greater deposition of extracellular matrix. It is known that TGFβ-1, the inducer of chondrogenesis used in this study, activates p38 kinase and also MAP kinase (MAPK) signaling cascades, including the activation of ERK [26–28]. Li et al. [26] showed that ERK and p38 are activated by TGFβ-1 in different patterns, with variation in their activity levels during chondrogenic differentiation protocol. In individuals with NF1, mutated neurofibromin is not able to properly inactivate Ras protein. Activated Ras continually activates numerous intracellular signaling pathways, such as p38 kinase and MAPK/ERK pathway [3]. Therefore, a possible explanation for the greater deposition of extracellular matrix found in our study after chondrogenic differentiation is the constant activation of Ras, which elevates p38 kinase and ERK levels during the entire process of chondrogenic differentiation. Further studies are needed to confirm this hypothesis.

Previous studies have shown that chondrocytes isolated and cultured on a 2D environment change their morphology and behavior [29]. Furthermore, it has been demonstrated that the importance of an in vitro 3D environment for the formation of hypertrophic chondrocytes in chondrogenic differentiation process, due to cell-matrix interactions [30, 31]. Since in vitro 3D environment better mimics the in vivo microenvironment, its use is important to better understand the alterations in the chondrogenesis process in NF1. In the present study, chondrogenic differentiation in pellet (3D) was confirmed by the presence of GAGs, using Alcian blue staining. It was also confirmed by the presence of matrix vesicles in both the semithin and ultrathin sections, and also by the presence of collagen fibers in TEM. Moreover, the absence of Ki-67 expression demonstrated that the cells had achieved the terminal stage of differentiation. This finding has already been demonstrated in human mesenchymal stem cells by Dexheimer et al. [32] who showed a decrease in cell proliferation as the chondrogenic differentiation occurred, with no longer proliferation at the end of the differentiation process.

The higher amount of collagen fibers in NF1 cultures pellets, observed through TEM, corroborates with our results of chondrogenic differentiation in 2D culture, in which there was an increase of extracellular matrix. In addition, in the 3D model, we also showed variability in the size of matrix vesicles in NF1, ranging from small to large sizes. These results demonstrate that chondrogenesis in NF1 is different when compared with the controls, and this may contribute to the orthopedic problems in these individuals. Studies with animal models have shown that neurofibromin is required for proper endochondral ossification and osteoblast differentiation [8, 9]. Mice lacking neurofibromin in osteochondroprogenitor (*Nf1*^−/−^) cells exhibited orthopedic problems in bones derived from endochondral ossification, e.g. shortening of skull base, maxilla and zygomatic bones [9]. Individuals with NF1 present short skull base, short mandible and maxilla, [33] as well as other alterations in stomatognathic system, [34–36] which have neural crest origin, the same embryonic origin of DPSCs. Therefore, it is possible that these bone alterations in individuals with NF1 are due to abnormalities in cartilaginous growth and poor osteogenesis.

We demonstrated that DPSCs, both in 2D and 3D culture models, can be used as an in vitro model for the study of chondrocyte alterations in NF1. The use of DPSCs may also be further explored to study other manifestations of NF1. For example, DPSCs are capable to differentiate into skeletal muscle cells and it is known that individuals with NF1 present reduced muscle function [37, 38] and that neurofibromin is essential for the correct function of the muscular system [39]. Moreover, endothelial cells differentiated from DSCPs could be used to investigate the endothelial alterations that occur in NF1. Individuals with NF1 have increased prevalence of cardiovascular diseases, including obstructive vascular disorders, [40] and an in vivo study showed that neurofibromin has an essential role in endothelial cells [41].

## Conclusion

NF1 cell cultures present increased matrix deposition during chondrogenic differentiation compared with controls, indicating an alteration in the process of chondrogenesis.

## Additional files

Additional file 1: Amount of stained area analyzed in the images obtained from each assay after chondrogenic differentiation. (DOCX 58 kb)

Additional file 2: A: Positive control (palatine tonsil) evidencing strong nuclear immunostaining in germinal centers cells; B-G: Absence of Ki-67 expression in the pellets after 21 days of chondrogenic differentiation (B, F: CT10, C: CT11, D: NF37, E, G: NF87). (TIF 13198 kb)

Additional file 3: Stained area analyzes after osteogenic and adipogenic differentiation. To obtain the percentage of stained area after osteogenic and adipogenic differentiation, five random images (20× magnification) were obtained from each triplicate and analyzed using ImageJ software. The percentage of stained calcium deposits in control cultures was significantly higher comparing with NF1 cultures (*p* < 0.0001; Mann-Whitney test) while no significant difference between NF1 and control cultures was found after adipogenic differentiation (*p* = 0.316, Mann-Whitney test). A: Osteogenic differentiation, B: Adipogenic differentiation. (TIF 1072 kb)

## Abbreviations

2D: Two-dimensional
3D: Three-dimensional
DMEM: Dulbecco’s Modified Eagle Medium Nutrient Mixture
DPSCs: Dental pulp stem cells
FBS: Fetal bovine serum
GAGs: Glycosaminoglycans
HE: Hematoxylin and eosin
MSCs: Mesenchymal stromal cells
NF1: Neurofibromatosis 1
PBS: Phosphate-buffered saline
SHED: Stem cells from human exfoliated deciduous teeth
TEM: Transmission electron microscopy
TGFβ-1: Transforming growth factor beta 1
