# Supplementary material for: Increased extracellular matrix deposition during chondrogenic differentiation of dental pulp stem cells from individuals with neurofibromatosis type 1: an in vitro 2D and 3D study
Source: Orphanet J Rare Dis. 2018 Jun 25;13:98. doi: 10.1186/s13023-018-0843-1 (PMC6020206; doi:10.1186/s13023-018-0843-1)
Supplement: Supplementary file 1 — Amount of stained area analyzed in the images obtained from each assay after chondrogenic differentiation. (DOCX 58 kb) [file 13023_2018_843_MOESM1_ESM.docx]

**Table 1.** Amount of stained area analyzed in the images obtained from each assay after chondrogenic differentiation.

| **Chondrogenic Differentiation** | | Value | Average per cell culture | Average per group | *P* |
| --- | --- | --- | --- | --- | --- |
| NF37 | Assay 1 | 51.07 | 62.89 | 64.01 | 0.004 |
|  | Assay 2 | 65.91 |  |  |  |
|  | Assay 3 | 54.72 |  |  |  |
|  | Assay 4 | 79.84 |  |  |  |
| NF87 | Assay 1 | 77.95 | 65.12 |  |  |
|  | Assay 2 | 59.87 |  |  |  |
|  | Assay 3 | 63.47 |  |  |  |
|  | Assay 4 | 59.21 |  |  |  |
| CT10 | Assay 1 | 36.31 | 50.62 | 47.76 |  |
|  | Assay 2 | 39.41 |  |  |  |
|  | Assay 3 | 62.1 |  |  |  |
|  | Assay 4 | 64.66 |  |  |  |
| CT11 | Assay 1 | 57.57 | 46.88 |  |  |
|  | Assay 2 | 45.57 |  |  |  |
|  | Assay 3 | 42.94 |  |  |  |
|  | Assay 4 | 41.46 |  |  |  |
| CT12 | Assay 1 | 40.33 | 45.77 |  |  |
|  | Assay 2 | 48.91 |  |  |  |
|  | Assay 3 | 50.67 |  |  |  |
|  | Assay 4 | 43.16 |  |  |  |
