# Supplementary figures and images for: Increased extracellular matrix deposition during chondrogenic differentiation of dental pulp stem cells from individuals with neurofibromatosis type 1: an in vitro 2D and 3D study
Source: Orphanet J Rare Dis. 2018 Jun 25;13:98. doi: 10.1186/s13023-018-0843-1 (PMC6020206; doi:10.1186/s13023-018-0843-1)

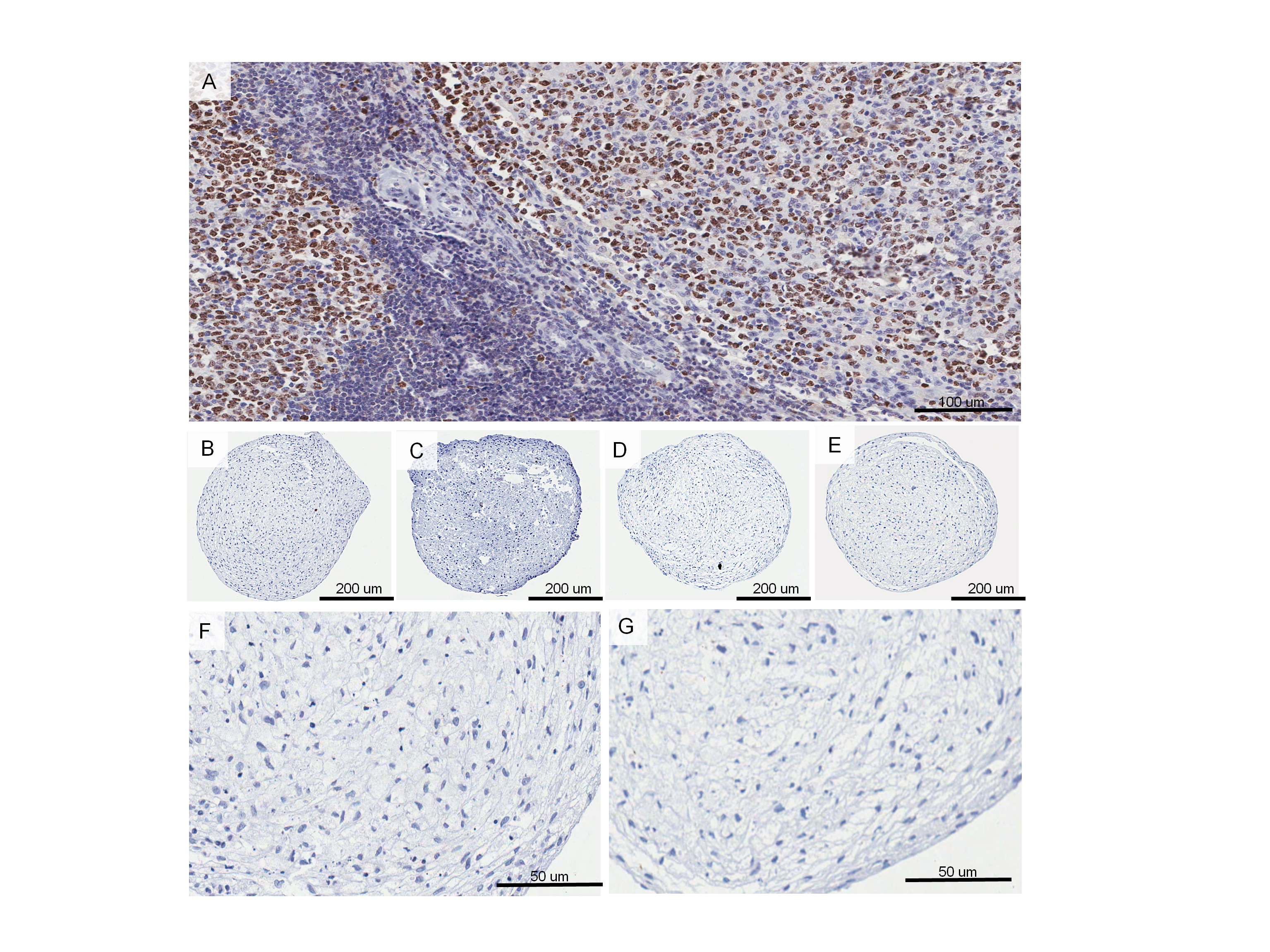

Supplement: Supplementary file 2 — A: Positive control (palatine tonsil) evidencing strong nuclear immunostaining in germinal centers cells; B-G: Absence of Ki-67 expression in the pellets after 21 days of chondrogenic differentiation (B, F: CT10, C: CT11, D: NF37, E, G: NF87). (TIF 13198 kb) [file 13023_2018_843_MOESM2_ESM.tif]

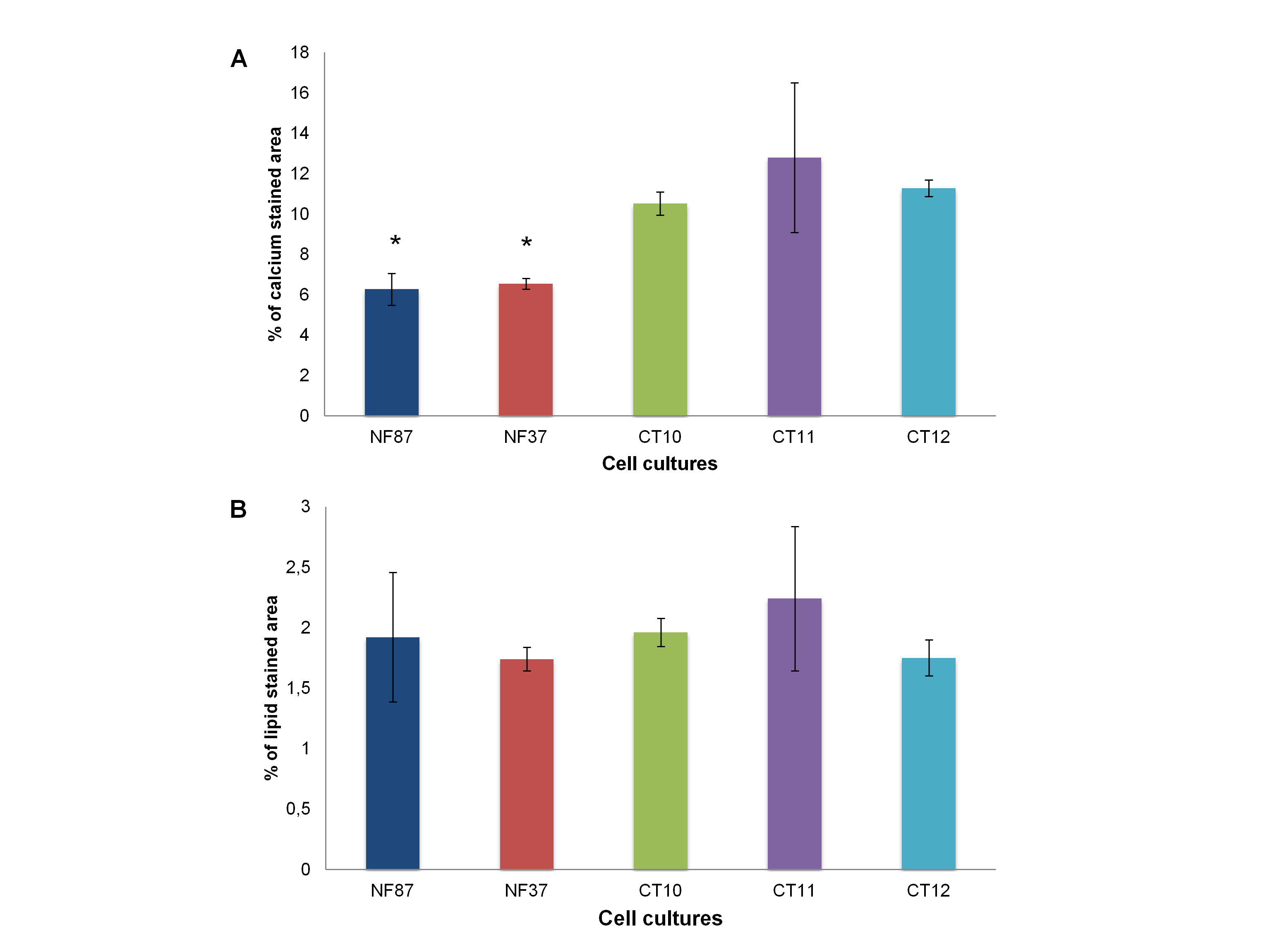

Supplement: Supplementary file 3 — Stained area analyzes after osteogenic and adipogenic differentiation. To obtain the percentage of stained area after osteogenic and adipogenic differentiation, five random images (20× magnification) were obtained from each triplicate and analyzed using ImageJ software. The percentage of stained calcium deposits in control cultures was significantly higher comparing with NF1 cultures (p < 0.0001; Mann-Whitney test) while no significant difference between NF1 and control cultures was found after adipogenic differentiation (p = 0.316, Mann-Whitney test). A: Osteogenic differentiation, B: Adipogenic differentiation. (TIF 1072 kb) [file 13023_2018_843_MOESM3_ESM.tif]
